# Editorial: Chemical Modification of Adsorbents for Enhanced Carbon Capture Performance

**DOI:** 10.3389/fchem.2021.657669

**Published:** 2021-03-15

**Authors:** Georgios N. Karanikolos, George Em. Romanos, Lourdes F. Vega

**Affiliations:** ^1^ Department of Chemical Engineering, Khalifa University, Abu Dhabi, United Arab Emirates; ^2^ Research and Innovation Center on CO_2_ and H_2_ (RICH), Khalifa University, Abu Dhabi, United Arab Emirates; ^3^ Center for Catalysis and Separations (CeCaS), Khalifa University, Abu Dhabi, United Arab Emirates; ^4^ Institute of Nanoscience and Nanotechnology (INN), Demokritos National Research Center, Athens, Greece

**Keywords:** carbon capture, adsorption, functionalization, CO_2_, carbon dioxide

Efficiently capturing carbon dioxide from diverse sources and under different conditions of temperature, pressure and composition is vital in mitigating the impact of continuously increasing CO_2_ levels into the atmosphere (Alivisatos and Buchanan, 2010; Rangaraj et al.). Of the various techniques, adsorption presents particular prominence mainly due to low energy consumption, cyclability, fast kinetics, versatility of potential adsorbent materials, range of operating conditions, and the fact that it is environmentally-friendly (Karousos et al., 2017; Bahamon et al., 2020; Varghese et al., 2020). In this collection, a variety of adsorbent families among the most promising ones for carbon capture were studied and optimized via various functionalization strategies, i.e., metal-organic frameworks (MOFs), zeolites, porous carbons, Deep Eutectic Solvent (DES)/carbon nanotube (CNT) systems, and graphene oxide adsorbents, as summarized below.

The contribution by Kamran et al. focused on heteroatom-rich porous carbon adsorbents derived from an inexpensive, non-toxic, and easily available polyacrylonitrile (PAN) precursor. The adsorption performance of the resulting activated carbons was optimized by parametrically varying the types of activators used at different temperatures. Namely, NaOH, KOH, K_2_CO_3_, and KNO_3_ were employed and the effect on textural properties and CO_2_ adsorption performance was evaluated. For the optimum adsorbents, high surface areas were achieved (>3,000 m^2^/g) combined with high pore volumes, features that resulted in enhanced CO_2_ capacities of up to 163 mg/g (NaOH-activated) at ambient conditions, coupled to high CO_2_/N_2_ selectivity. In addition, the heat of adsorption of the developed adsorbents was relatively low (<40 kJ/mol) indicating a physisorption mechanism, which can ensure a small amount of energy required for regeneration, and the possibility of a PSA-based capture process. Indeed, efficient cyclic operation of the developed porous carbons was also demonstrated in the paper.

Thomou et al.performed silylation of organically modified graphene oxide using various silica precursors (APTEOS, BTB, and TEOS) aiming at creating high-yield silica networks as pillars between the organo-modified graphene oxide (GO) layers. Thermal decomposition of the organic moieties endowed the pillared GO with enhanced porosity and resulted in robust 3D structures. XPS and FTIR spectroscopy evidenced reduction of GO to graphene after calcination of the heterostructures and confirmed the presence of silica. The resulting porous materials had a sponge-like structure, with a BET surface area of 550 m^2^/g in the case of G-BTB, and exhibited a CO_2_ adsorption capacity of up to 3.5 mmol/g at 5 bar and 0°C, paving the way for further studies on this type of approach to generate novel porous heterostructured graphene-based adsorbents.

Deep eutectic solvents were employed in the work by Zaib et al. to achieve functional dispersions of CNTs as to promote disaggregation and enhance the carbon dioxide capture performance. Using single walled carbon nanotubes (SWNTs) and Choline Chloride-Glycerol (ChCl-Gly) as DES, the authors explored the effect of critical parameters on the final dispersions, i.e. DES concentration in water, sonication energy upon dispersion formation, and concentration of the SWNTs. The impact of the dispersion factors was quantified by determining the average aggregate diameter and the polydispersity index (PDI) of the SWNTs in the aqueous DES systems. Equilibrium thermodynamics and quantum chemistry modeling was also applied, experimentally verified, and statistically validated to map the impact of these factors and to obtain optimized dispersions. Indicatively, optimized dispersions were characterized by small (<100 nm) and uniform (<0.1 PDI) SWNT aggregates obtained at relatively low sonication energy. Such approach is a significant step forward toward development of hybrid (slurry) adsorption/absorption systems, taking advantage of multiple sorption mechanisms for efficient CO_2_ capture.

Zeolite-based adsorbents and in particular aluminophosphates (AlPO_4_-5) were explored in the work by Papageorgiou et al. Parametric optimization of these adsorbents was achieved by studying the effect of ion substitution (Fe, Mg, Co., and Si) into the AFI framework, employing different activation methods (calcination and pyrolysis), and manipulating the reaction mixture dilution rate and the resulting crystal morphology. Upon activation by pyrolysis in inert atmosphere, low temperature treatment (240°C) enhanced CO_2_ interaction with the surface at low pressures (up to 1 bar) due to the existence of remnant carbon species in the pores from the partial decomposition of the structure directing agent (SDA). Among the ion-substituted analogues, FeAPO-5 was found to exhibit the highest CO_2_ capacity at all tested pressures (up to 4 bar). Optimization of the synthesized FeAPO-5 adsorbents led to the material that contained the minimum portion of extra-framework or clustered iron and the highest mesoporosity (Fe/Al_2_O_3_ molar ratio of 5). Furthermore, low water content in the synthesis gel led to the formation of spherical agglomerates of small 2D-like crystallites that exhibited higher adsorption capacity compared to columnar-like crystals produced by employing more diluted mixtures. Fast CO_2_ adsorption kinetics were also obtained from the optimum materials that followed a pseudo–first-order model. The isosteric head of adsorption was low (<25 kJ/mol) rendering these materials promising for PSA capture processes.

Amine functionalization of MOFs was explored in the work by Bahamon et al. by means of Grand Canonical Monte Carlo (GCMC) molecular simulations. Different types of amine-functionalized MOFs were analyzed focusing on optimization for post-combustion CO_2_ capture. Specifically, six amine models of different chain lengths and degree of substitution grafted to the unsaturated metal sites of the M_2_(dobdc) MOF (MOF-74) and its expanded version, M_2_(dobpdc), were evaluated in terms of adsorption isotherms, selectivity, cyclic working capacity and regenerability, and compared to experimental data. Among the combinations explored, mmen-Mg/DOBPDC and mda-Zn/DOBPDC showed high cyclic working capacities upon temperature swing adsorption (TSA). The -mmen functionalized structure in particular showed higher CO_2_ uptake and better regenerability for the flue gas mixtures and conditions studied. The work also explored the negative effect of steric hindrance and set the associated limits beyond which more amine functional groups grafted on the MOFs and/or full functionalization of the metal centers did not lead to better CO_2_ separation capability. Such calculations can shed light on how functionalization can enhance gas adsorption via the cooperative chemi-physisorption mechanism of these materials, and how the materials can be tuned to achieve desired adsorption characteristics customized to the conditions of the CO_2_-containing mixture to be processed.
